# Occurrence, fate, and risk assessment of antibiotics in typical pharmaceutical manufactories and receiving water bodies from different regions

**DOI:** 10.1371/journal.pone.0270945

**Published:** 2023-01-20

**Authors:** Yuanfei Liu, Dan Cai, Xin Li, Qingyao Wu, Ping Ding, Liangchen Shen, Jian Yang, Guocheng Hu, Jinhua Wu, Lijuan Zhang

**Affiliations:** 1 School of Environment and Energy, South China University of Technology, Guangzhou, Guangdong, China; 2 State Environmental Protection Key Laboratory of Environmental Pollution Health Risk Assessment, South China Institute of Environmental Sciences, Ministry of Ecology and Environment, Guangzhou, Guangdong, China; 3 School of Public Health and Emergency Management, South University of Science and Technology of China, Shenzhen, Guangdong, China; Sam Higginbottom University of Agriculture, Technology and Sciences, INDIA

## Abstract

This study aimed to investigate the presence and persistence of antibiotics in wastewater of four typical pharmaceutical manufactories in China and receiving water bodies and suggest the removal of antibiotics by the wastewater treatment process. It also evaluated the environmental impact of antibiotic residues through wastewater discharge into receiving water bodies. The results indicated that thirteen antibiotics were detected in wastewater samples with concentrations ranging from 57.03 to 726.79 ng/L. Fluoroquinolones and macrolides were the most abundant antibiotic classes found in wastewater samples, accounting for 42.5% and 38.7% of total antibiotic concentrations, respectively, followed by sulfonamides (16.4%) and tetracyclines (2.4%). Erythromycin-H_2_O, lincomycin, ofloxacin, and trimethoprim were the most frequently detected antibiotics; among these antibiotics, the concentration of ofloxacin was the highest in most wastewater samples. No significant difference was found in different treatment processes used to remove antibiotics in wastewater samples. More than 50% of antibiotics were not completely removed with a removal efficiency of less than 70%. The concentration of detected antibiotics in the receiving water bodies was an order of magnitude lower than that in the wastewater sample due to dilution. An environmental risk assessment showed that lincomycin and ofloxacin could pose a high risk at the concentrations detected in effluents and a medium risk in their receiving water bodies, highlighting a potential hazard to the health of the aquatic ecosystem. Overall, The investigation was aimed to determine and monitor the concentration of selected antibiotics in 4 typical PMFs and their receiving water bodies, and to study the removal of these substances in PMFs. This study will provide significant data and findings for future studies on antibiotics-related pollution control and management in water bodies.

## Introduction

Antibiotics are natural, synthetic, or semi-synthetic compounds that can kill or inhibit the growth or metabolic activity of microorganisms. These compounds are biologically active molecules with antibacterial, antifungal, and antiparasitic properties. They have been widely used to treat infectious diseases in humans and animals, and have benefited by treating infections in livestock farms, aquaculture, and agriculture [1]. The data from 76 countries showed that the total global antibiotic consumption increased from 21.1 to 34.8 billion defined daily doses between 2000 and 2015 [2]. Studies have shown that antibiotics are detected in surface water [3, 4], groundwater [5, 6], domestic sewage [5, 7, 8], sediment [6, 9, 10], soil [11, 12], and even drinking water [13, 14] indicating that the environmental antibiotic pollution is widespread. In addition, the concentrations of antibiotics in Asian developing countries tend to be higher than those generally reported in European and North American countries [15]. It is estimated that more than 70 antibiotics with concentrations up to several micrograms per liter have been detected in 7 major water systems in China (Yin et al., 2021) [16]. Importantly, long-term exposure to antibiotics promotes the development of antibiotic-resistant bacteria (ARB) and antibiotic-resistant genes (ARG), thereby reducing the therapeutic potential against bacterial pathogens. According to the World Health Organization (WHO), ARGs are considered one of the major threats to human and animal health in the 21st century [9, 17–21]. Therefore, a comprehensive understanding of the environmental emission and fate and risk of antibiotics in water bodies is essential.

The primary sources of antibiotics in the environment are municipal wastewater treatment plants, agricultural settings, aquaculture, hospitals, and pharmaceutical production facilities [15]. Pharmaceutical manufactories (PMFs) have been proposed as important reservoirs of antibiotics. In most cases, the wastewater is discharged after treatment in the wastewater treatment facility of PMFs. However, antibiotic residues cannot be removed completely by the existing wastewater treatment process of PMFs. Research in Taiwan found that PMFs were an important source of antibiotics, with sulfamethoxazole having a maximum concentration exceeding 1,000,000 ng/L [22]. Pakistan reported a high level of antibiotic residues in wastewaters close to the pharmaceutical factories and a positive correlation between the level of residues and antibiotic resistance in the samples [23]. Many researches have studied that the concentration levels of antibiotics detected in sewage treatment plants (STPs) might threaten the nontarget organisms, such as algae, *Daphnia magna*, mollusks, and fish [24–27]. However, current studies have mostly focused on removing antibiotics from STPs or the determination of antibiotics in natural water bodies, the occurrence, fate and environmental risk of antibiotics during wastewater treatment processes at PMFs are not well understood yet [28].

China’s pharmaceutical industry is developing rapidly and has grown to be the second largest pharmaceutical market in the world [29], reaching 373 billion dollar in 2018. Many of the PFMs are registered to produce antibiotic products. The majority of registered antibiotic products belongs to groups of quinolones (norfloxacin, ofloxacin, ciprofloxacin and so on), macrolides (neomycin, clarythromycin, azithromycin and so on), sulfonamides(sulfamethoxazole, sulfadiazine, sulfamethazine and so on), and tetracyclines(tetracycline, oxytetracycline, chlortetracycline and so on). In recent years, more and more studies have focused on the effect of removal of antibiotics in STPs and the effluent from STPs on antibiotics in receiving water, but few studies have investigated the effect of control for antibiotics in PMFs and the effluent from PMFs on antibiotics in receiving water bodies [4, 28, 30, 31]. In this study, We investigated the occurrence and fate of antibiotics in wastewaters from 4 typical PMFs of Hebei, Jiangsu, Zhejiang, Guangdong, China. The presence of antibiotics were also monitored in their receiving water bodies, from upstream of the river to the wastewater discharge point and downstream of the discharge point, to assess the occurrence and impact of these antibiotics in these rivers. Finally, the potential ecological risks of the target antibiotics to aquatic species were assessed according to the calculated risk quotients (RQs). The results of this study provide systematic and detailed insight into the removal efficiency and control of antibiotics by PMFs, as well as the impact of pollutants in the effluent water of PMFs on the receiving water bodies. Moreover, this study provides subsequent data for further studies on developing water pollution treatment and pharmaceutiacl manufactory treatment processes. The findings also provided important background data for the pollution control of antibiotics in the aquatic environment of study areas.

## Materials and methods

### Materials and reagents

The 17 target antibiotics belonging to 4 different classes included the following: (I) 1 tetracycline (TC), metacycline (MTC); (II) 9 sulfonamides (SAs), sulfachlorpyridazine (SCP), sulfadiazine (SDZ), sulfadimethoxine (SDM), sulfamethazine (SMZ), sulfameter (SME), sulfamethoxazole (SMX), sulfamonomethoxine (SMM), sulfapyridine (SPD), and trimethoprim (TMP); (III) 4 macrolides (MLs), clarithromycin (CTM), erythromycin-H_2_O (ERY-H_2_0), lincomycin (LIN), and roxithromycin (ROX); and (IV) 3 fluoroquinolones (FQs), ciprofloxacin (CIP), norfloxacin (NFX), and ofloxacin (OFL), All the antibiotics standards were of high purity grade [high-performance liquid chromatography (HPLC) grade, >99%] and purchased from Anpel (Shanghai, China). Isotopically labeled compounds, including sulfamethoxazole-D_4_ (SMX-D_4_), erythromycin-^13^C-D_3_ (ERY-^13^C-D_3_), thiabendazole-D_4_ (TBD-D_4_), ciprofloxacin-D_8_ (CFX-D_8_), sulfamethazine-^13^C_6_ (SMZ-^13^C_6_), trimethoprim-D_3_ (TMP-D_3_) and lincomycin-D_3_ (LIN-D_3_), were obtained from Cambridge Isotope Laboratories (England) and C/D/N Isotopes (Canada).

Reagent-grade methanol, acetonitrile, formic acid, and other chemicals were purchased from local suppliers. Milli-Q water was used throughout the study. The stock solution of each antibiotic class was prepared in methanol. Working solutions with different concentrations were prepared by mixing and diluting the stock solutions.

### Sample collection and preparation

**Fig 1:**
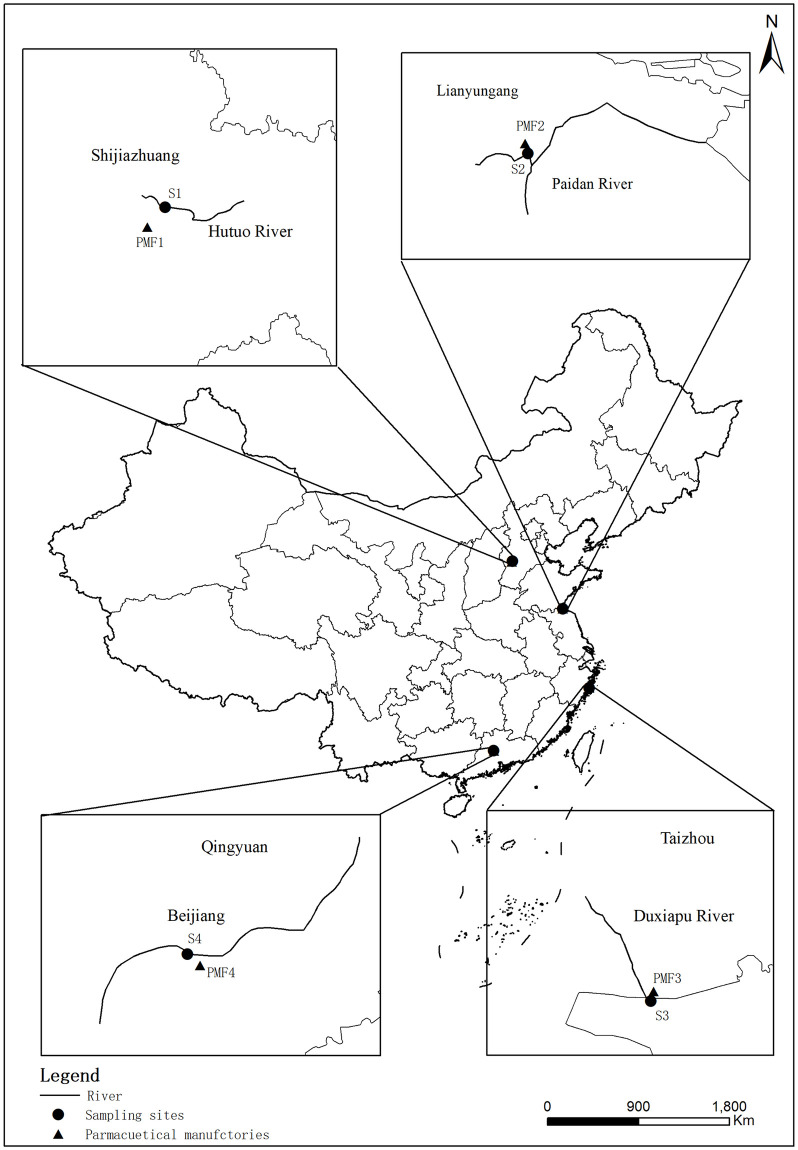
Sampling locations of the four PMFs (PMF 1 –PMF 4) and other sites in China.

**Fig 2:**
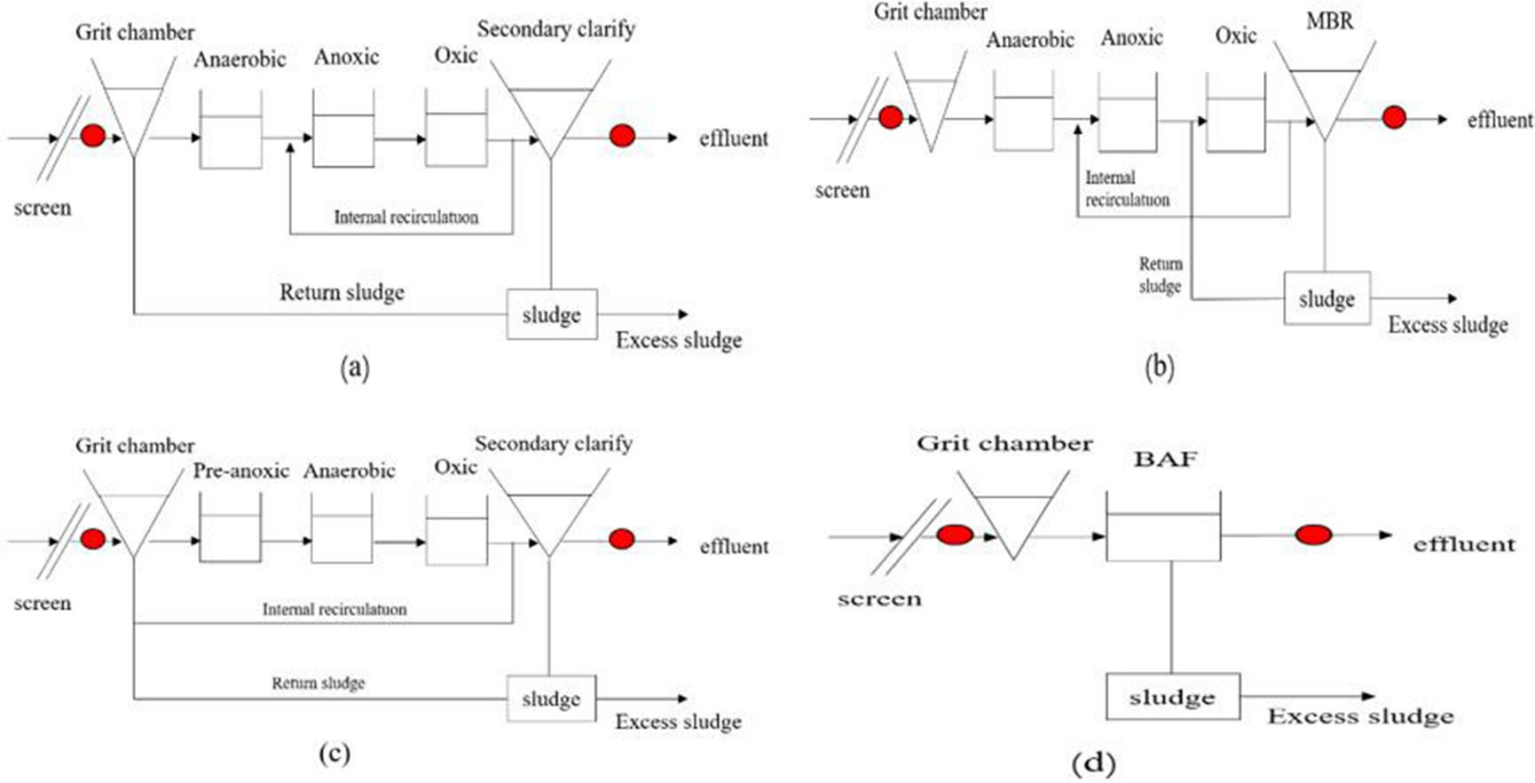
Flow charts of technological processes and sampling sites of four PMFs. (a) PMF1, A^2^O, (b) PMF2, MBR, (C) PMF3, modified AO, and (d) PMF4, BAF.

This research was conducted under the National Key Research and Development Program (2018YFC1801505) and was approved and supported by the Ministry of Science and Technology of the People’s Republic of China(MOST). The wastewater samples were collected from the influent and effluent of four PMFs, as well as their receiving water bodies in Hebei, Jiangsu, Zhejiang and Guangdong province of China. PMF 1 is located in Shijiazhuang City, Hebei Province, at the right bank of the Hutuo River (site PMF 1, 38° 1′ 27.77′ N; 114° 40′ 43.32′ E), which is the main tributary of the urban river in Shijiazhuang City, collecting urban domestic sewage and industrial wastewater; PMF 2 is located in Lianyungang City, Jiangsu Province, along the Paitan River(site PMF 2, 34° 39′ 46.87′ N; 119° 12′ 31.32′ E), producing antibiotics and other APIs; PMF 3 is an important chemical raw material pharmaceutical base in Linhai City, Zhejiang Province, located along the Du Xia Pu River(site PMF 3, 28° 41′ 48.19′ N; 121° 33′ 3.6′ E); PMF 4 is a typical pharmaceutical enterprise in Qingyuan City, Guangdong Province, mainly producing macrolide antibiotics, located along the Beijiang River(site PMF 4, 23° 40′ 25.32′ N; 113° 4′ 24.24′ E), which is a major urban river in Qingyuan, collecting urban domestic and industrial wastewater. The specific sampling locations are depicted in Fig 1. Eight sampling points are georeferenced in S1 Table. The basic information of each PMF is shown in S2 Table. Different sewage treatment technologies used in the four PMFs included Anaerobic–Anoxic–Oxic (A^2^O), Membrane Bio-Reactor (MBR), Anaerobic–Oxic (modified AO), Biological Aerated Filters (BAF). The process flow charts and sampling sites are shown in Fig 2. Wastewater samples were collected from the influent and effluent of PMFs. Besides, the surface water from the upstream/downstream of the receiving water bodies (100 m away from the effluent outfall) was also collected. All the samples in three replicates were obtained during November–December 2020 and July 2021. The samples from the PMFs were collected in 1-L amber glass as 24-h composite, while the samples from receiving water bodies were collected as grab samples in the middle of the day. All samples were placed in a cool place at –20°C and analyzed within 7 days to minimize degradation. Each analysis was repeated three times, and the reported results were based on the average value.

### Quantification of antibiotics

The concentration of target antibiotics in wastewater samples was measured using solid-phase extraction (SPE) combined with liquid chromatograph coupled to tandem mass spectrometry (LC-MS/MS, Agilent Liquid Chromatography 1260 coupled to an AB SCIES API-4000 triple quadrupole MS) under multiple reaction monitoring (MRM) conditions with positive electrospray ionization (ESI) mode, as described in our previous studies [32] and based on the Environmental Protection Agency guidelines [33].

Briefly, 250 mL of wastewater samples (pH 3.0) was first spiked with isotopically labeled internal standards, including SMX-D_4_, ERY-^13^C-D_3_, TBD-D_4_, CFX-D_8_, SMZ-^13^C_6_, TMP-D_3_, LIN-D_3_. Then, the spiked samples were passed through a pre-conditioned SPE cartridge for extraction (S1 Text). In the extracts, the target antibiotics were separated using a C18 column (Agilent ZORBAX Eclipse Plus, 100 mm × 2.1 mm, 1.8 μm) during LC-MS/MS analysis. The mobile-phase solutions were 0.2% formic acid and 2 mM ammonium acetate (A) and acetonitrile (B). The antibiotics were quantified in positive ESI mode. The LC gradient program for antibiotic separation and full MS/MS measurement conditions for the individual compound are reported in S3 Table.

### Quality assurance and quality control (QA/QC)

The concentrations of the target antibiotics in the samples were performed using internal standard method. And the data generated from the analysis were subject to strict quality control procedures. Procedural blanks and parallel samples (one per ten samples) were inserted during all testing as a regular part of the analysis. Results in the field and procedural blanks for all analytes were below the limit of detection (LOD). The internal standard method was used for quantification with standard curves of 12 points ranging from 0.1 ng/mL to 500 ng/mL. The coefficients (R2) for all target analytes were over 0.99. All of the target antibioticswere below the LOD in all blank samples (i.e., Milli-Q water). The analytical quality parameters LOD, limit of quantification (LOQ), and recovery values (%) are listed in S4 Table. The LOD ranged from 0.16 ng/L to 1.78 ng/L, whereas LOQ ranged from 0.52 to 5.88 ng/L. Concerning the extraction methodology, the recoveries achieved for all target extracted compounds ranged between 60% and 130%.

### Statistical analysis

The statistical summary of experimental data was completed in Excel 2013. The graphics were drawn using OriginPro 2021, and the correlation analysis was completed in IBM SPSS 26.

### Risk assessment

In this study, the potential ecological risks of target antibiotics to aquatic ecosystems were assessed by calculating the RQs of antibiotics in final wastewater and receiving water bodies [34]

$$\text{RQ}=\frac{\text{MEC}}{\text{PNEC}}\text{PNEC}=\frac{{\text{LC}}_{50}}{\text{AF}}\text{or}\frac{{\text{EC}}_{50}}{\text{AF}}\text{or}\frac{\text{NOEC}}{\text{AF}}$$

where MEC is the measured maximum environmental concentration (ng/L) of the target compound, and PNEC is the predicted no-effect concentration (ng/L) of the target compound in the water body. The information on PNECs were obtained from previously published works (S5 Table). The environmental risk factors were divided into four grades according to the RQ value of individual antibiotics: insignificant (RQs < 0.01), low risk (0.01 ≤ RQs ≤ 0.1), intermediate risk (0.1 ≤ RQs ≤ 1.0), and high risk (RQs > 1.0) [35, 36].

## Result and discussion

### Presence of antibiotics in raw influents and treated effluents from PMFs

Seventeen antibiotics were investigated in the influents and effluents of four PMFs in Hebei, Jiangsu, Zhejiang, and Guangdong provinces. Of the 17 selected antibiotics, 13 were detected in wastewater samples from all PMFs, including 5 SAs (SMZ, SD, SMX, SPD, and TMP), 4 MLs (CTM, ERY, LIN, and ROX), 3 FQs (CIP, NOR, and OFL) and 1 TC (MTC). Their detection frequencies in all samples are shown in S6 Table. ERY and OFL were the antibiotics of the highest detection frequency (75%). TMP, LIN, and ROX occurred with a detection frequency of more than 50%. Among the wastewater samples investigated, the influents had a higher detection frequency (76.5%) than effluents (64.7%), implying the effluent from PMFs was an important source of these compounds to the receiving water bodies in the area, and the reduced two antibiotics may have been removed completely after passing through the wastewater treatment facility of the pharmaceutical manufactory. Overall, the high detection frequencies suggested the widespread existence of antibiotics in the PMFs from Hebei, Jiangsu, Zhejiang, and Guangdong, China.

The concentrations of detected antibiotics were in the range of 125.49ng/L-403.9ng/L in raw influent, 57.03ng/L-726.79ng/L in treated effluent. Among them, the antibiotics belonging to MLs classes were most frequently detected in the wastewater samples The SAs and QNs classes followed, and only few TCs compounds were occasionally observed. The mean concentrations of the 13 antibiotics in four PMFs in influent ranged from 0.06 (SMZ) to 56.89 ng/L (OFL). The maximum concentrations of 6 antibiotics were detected in PMF 1, including CTM (3.82ng/L), ERY-H_2_O (41.31ng/L), NOR (109.01ng/L), OFL (179.03ng/L), ROX (68.26ng/L), and SD (1.92ng/L), inferring the relatively large production of antibiotics in this area. However, the maximum concentrations of the three antibiotics in the effluent samples were detected in PMF 2 suggesting the low removal efficiency of PMF 2.

### Composition patterns profile and regional distribution of antibiotics in PMFs

**Fig 3:**
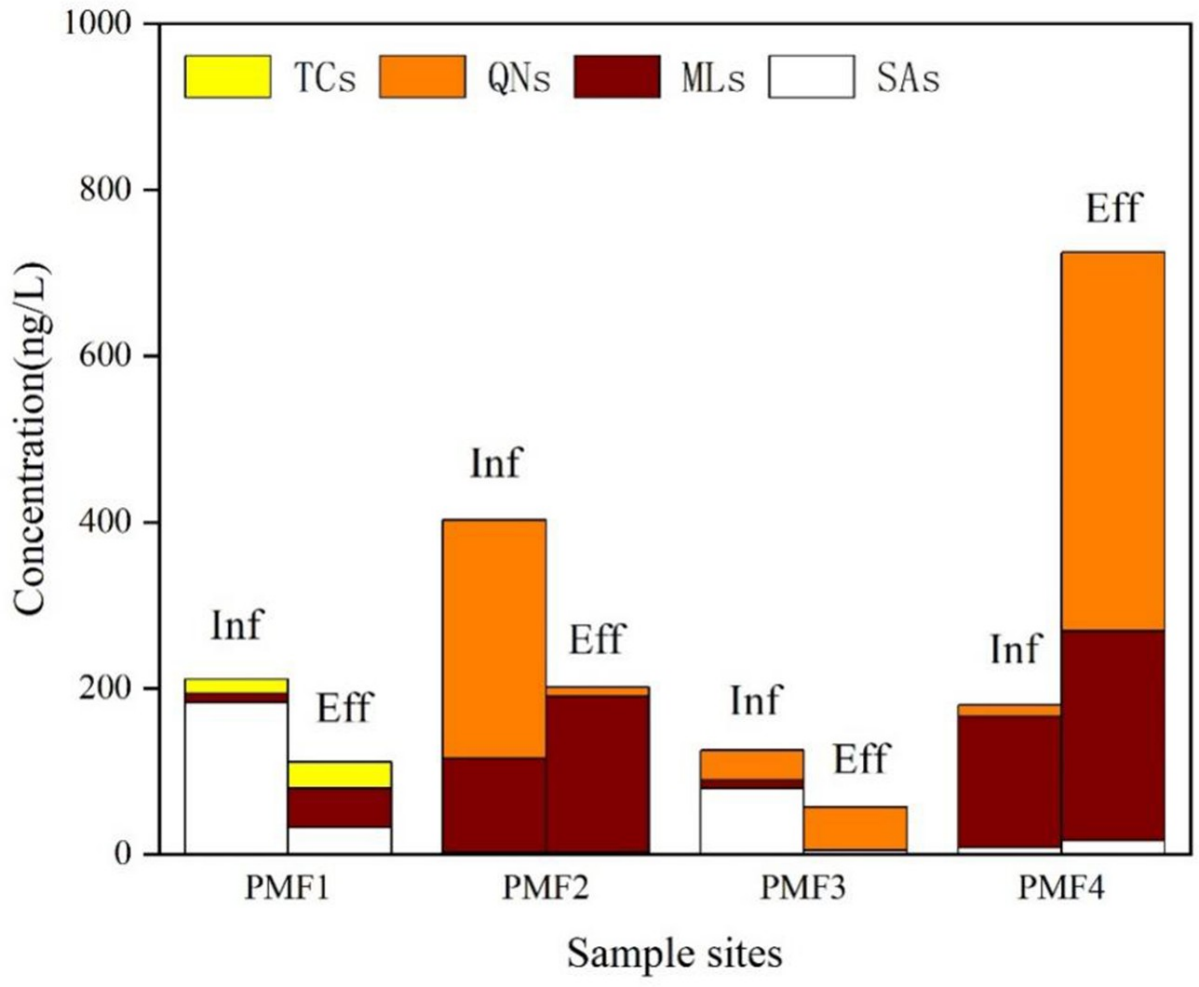
Concentrations of 13 antibiotics belonging to four antibiotic classes in the influent and effluent samples of 4 PMFs. Eff, effluent samples; Inf, influent samples.

The presence of antibiotics in effluents of PMFs exhibited imbalanced regional distribution (Fig 3). PMF 3 had the highest concentrations of total antibiotics (404.35 ng/L) and individual antibiotics (such as OFL, 179.03 ng/L) in the influent, possibly due to its large production scale. The total concentration of antibiotics in effluents of PMF 1 was comparable with that of the other two PMFs (PMFs 2 and 4). A high total concentration of antibiotics (about 800 ng/L) was found in PMF 4. The concentration of the main types of antibiotics varied significantly across regions. The composition patterns of antibiotics in each PMF are summarized in Fig 3. The apparent difference in antibiotic compositions in the influent and effluent waters was observed across PMFs (Table 1). The proportion of antibiotics in four PMFs ranged from 0% (TC in PMFs 1–3) to 88% (ML in PMF2). FQ accounted for the largest proportion of 71.3%. In the influent of PMF1, OFL and NOR were the main compounds whose concentrations reached 179.03 and 109.01 ng/L, respectively. MLs accounted for the largest proportion of 93.4% in the effluent of PMF1; clarithromycin and anhydrous erythromycin were the main compounds, with a concentration of 45.26 and 100.53 ng/L, respectively.

**Table 1:** Concentration (ng/L) and removal efficiency (%) of antibiotics in four PMFs samples (ng/L) (n = 16).

Compound	PMF 1		PMF 2		PMF 3		PMF 4	
	Influent	Effluent	Influent	Effluent	Influent	Effluent	Influent	Effluent
CIP	nd^a^	nd	nd	nd	4.71	33.58	nd	nd
CTM	nd	nd	3.82	45.26	nd	nd	nd	nd
ERY-H_2_O	10.49	31.81	41.31	100.53	9.01	nd	19.24	nd
LIN	nd	15.3	nd	nd	3.60	nd	133.05	252.33
NOR	nd	nd	109.01	nd	nd	nd	nd	nd
OFL	nd	nd	179.03	10.47	35.12	18.77	13.41	455.71
ROX	nd	nd	68.26	42.04	nd	nd	5.59	1.94
MTC	16.87	31.76	nd	nd	nd	nd	nd	nd
SD	nd	nd	2.92	3.71	nd	nd	nd	nd
SMZ	nd	nd	nd	nd	2.5	nd	nd	nd
SMX	159.15	24.02	nd	nd	nd	nd	nd	nd
SPD	nd	nd	nd	nd	39.21	3.77	nd	nd
TMP	24.22	8.6	nd	nd	39.91	nd	8.43	16.81

^a^ Not detected.

The proportion of MLs in the influent of PMF 2 was the highest. Among MLs, LIN was the predominant compound whose concentration was 133.05 ng/L; FQ and ML were the most abundant antibiotic classes in effluent samples of PMF2, accounting for 62.7% and 34.7% of the total antibiotic concentration, respectively. OFL and LIN were the main compounds, and their concentration reached 455.71 and 252.33 ng/L, respectively. The concentration of these two types of antibiotics in the effluent was much higher than that in the influent, which might be due to the influence of the hydraulic retention time, and both the influent and effluent waters were not from the same batch of water samples. SAs accounted for the largest proportion of 63.2% in the influent of PMF3; SPD and TMP were the main compounds, with a concentration of 39.21 and 39.91 ng/L, respectively. FQs accounted for the largest proportion of 91.8% in the effluent of PMF3; CIP was the main compound, with a concentration of 33.58 ng/L. SAs accounted for the largest proportion of 75.5% in the influent of PMF4; SMX was the main compound, with a concentration of 159.15 ng/L. MLs accounted for the largest proportion of 42.3%; dehydrated ERY-H_2_O and LIN were the main compounds, with a concentration of 31.81 and 15.3 ng/L, respectively.

The aforementioned findings revealed that the concentrations of TCs, administered by humans, and other antibiotics were extremely low in the recipient water bodies of the study areas, while FQs, MLs, and SAs were the main pollutants in PMFs. Sim et al. investigated the concentration of antibiotics in wastewater treatment facilities of four pharmaceutical factories in Busan, South Korea, and found that FQs and MLs were more than three orders of magnitude higher than those in this study [37]. In the wastewater treatment facilities of PMFs in Beijing, the detected mass concentrations of TCs and SAs in influents and effluents were more than three orders of magnitude higher than those in this study (Table 2) [38].

**Table 2:** Comparison of the antibiotic concentration levels in wastewater samples between this study and previous studies.

Compound	PMF		
	This study^a^ (ng/L)	Other studies (ng/L)	Country/Regions
CIP	Influent: <LOQ^b^-1.38 Effluent: <LOQ-33.58	Effluent: 28,000–31,000 × 10^3^	Sweden [39]
CTM	Influent: <LOQ-4.93 Effluent: <LOQ-45.26	Effluent: 40–800	Vietnam [40]
ERY-H_2_O	Influent: <LOQ-1.38 Effluent: <LOQ-33.58	Influent: 30.0–750.0 Effluent: <LOQ	Tianjin, China [41]
LIN	Influent: nd^c^–133.05 Effluent: nd–252.33	Effluent: Up to 35,538:	Shanghai, China [42]
NOR	Influent: nd–109.01 Effluent: nd	Effluent: 390–420×10^3^	Sweden [39]
OFL	Influent: nd–179.03 Effluent: nd–455.71	Effluent: 150–160×10^3^	Sweden [39]
ROX	Influent: nd–68.26 Effluent: nd–42.04	Influent: <10–375.3 Effluent: <LOQ	Tianjin, China [41]
MTC	Influent: nd–16.87 Effluent: nd–31.76	-^d^	-
SD	Influent: nd–2.92 Effluent: nd–3.71	Effluent: 3.0–20.0	Croatia [43]
SMZ	Influent: nd–2.5 Effluent: nd	Effluent: 6.7–231.0	Croatia [43]
SMX	Influent: nd–159.15 Effluent: nd–24.02	Effluent: Up to 50	Vietnam [40]
SPD	Influent: nd–39.91 Effluent: nd	-	-
TMP	Influent: nd–39.21 Effluent: nd	Effluent: 1–10 × 10^3^	India [44]

^a^ The range of minimum to maximum concentrations of antibiotics detected in the wastewaters.

^b^ Below the limit of quantification (LOQ) of analytical method.

^c^ Not detected.

^d^ Data were not found in literature.

### Removal of antibiotics in PMFs

Significant amounts of antibiotics were found in the PMF effluents, and the removal efficiency of the total antibiotics varied across four PMFs. We divided wastewater treatment plants of PMFs into four groups depending on the secondary treatment facilities to investigate the performance of different PMFs against the adopted treatment processes. The four PMFs applied different treatment processes: A^2^O, MBR, AO, and BAF. In this study, the removal efficiency of antibiotics in PMFs 1–3 (50.62%) using the secondary treatment technique such as cyclic activated sludge technology (CAST) was higher than those in PMF4 (–304.4%). The SAs were mostly removed (up to 100%) from the influents of PMFs. The removal efficiency of 90% could be achieved for FQs (e.g., NOR and OFL) in the PMFs. MLs were partially removed (–203.2%–100%), and TCs could not be found in most PMF effluents.

The removal efficiency varied from –1084.8% to 100% for each detected antibiotics separately in four PMFs (Table 3). Besides the treatment processes such as A^2^O, MBR, and AO applied by the PMFs, the BAF process effectively removed most detected antibiotics. NFX and OFL were rapidly dissipated by AAO, MBR, and AO treatment processes from the dissolved phase with the overall removal of 100% and 70.4%, respectively, with the exception of BAF technology. The findings were similar to those in the PMFs from Qingdao, China (93.9%) [45]. The high removal rate of FQs might be attributed to their negative charge under acidic conditions and strong adsorption capacity of the sludge. SPD and SMX were the second highest in terms of average removal efficiency of 90.3% and 89.6%, respectively. The high removal rate of SAs in this study contradicts the results of previous studies, with the removal efficiency of SMX (–31.2%–1.8%) in the wastewater of PMFs from China [45]. The negative removal efficiency of MLs (e.g., CTM, ERY-H_2_O, LIN) was observed in all PMFs. Given that the main route of excretion of MLs is through bile and feces, we can infer that fecal material is digested during biological treatment, thus increasing the dissolved mass load in the effluent observed in the present study [46].

**Table 3:** Overall removal efficiencies of the detected antibiotics in the wastewaters.

	Analyte	Removal efficienciesa in PMFs				
		Range (%)	PMF 1	PMF 2	PMF 3	PMF 4
SAs	SDZ	-41.2	nd^b^	-41.2	nd	nd
	SMZ	nd–100	nd	nd	100	nd
	SMX	nd–84.9	84.9	nd	nd	nd
	SPD	nd–90.3	nd	nd	90.3	nd
	TMP	–99–100	64.5	100	98.9	–99.4
MLs	CTM	–1084.8	nd	–1084.8	nd	nd
	ERY-H_2_O	–203.2–100	–203.2	–143.4	95.1	100
	LIN	–89.7–100	–79.2	nd	100	–89.7
	ROX	38.4–65.3	nd	38.4	nd	65.3
FQs	CIP	–612.9	nd	nd	–612.9	nd
	NFX	100	nd	100	nd	nd
	OFL	–3298.3–94.2	nd	94.2	46.6	–3298.3
TCs	MTC	–88.3	–88.3	nd	nd	nd
	Total	-304.4–58.14	47.09	50.04	58.14	-304.40

^a^ *$\text{Removal}\;\text{efficiencies}\;\left(\%\right)=\frac{{\text{C}}_{\text{influent}}-{\text{C}}_{\text{effluent}}}{{\text{C}}_{\text{influent}}}\times 100\%$*

^b^ No data

In addition, the removal efficiencies of the four processes for total antibiotics showed that the A^2^O, AO and MBR processes were not significantly different but significantly higher than the BAF process for the removal of target antibiotics from wastewater, which could be attributed to different treatment techniques (AS and CAST), operational parameters (hydraulic retention timeand temperature), and so forth.

### Occurrence of the selected antibiotics in receiving water bodies

**Fig 4:**
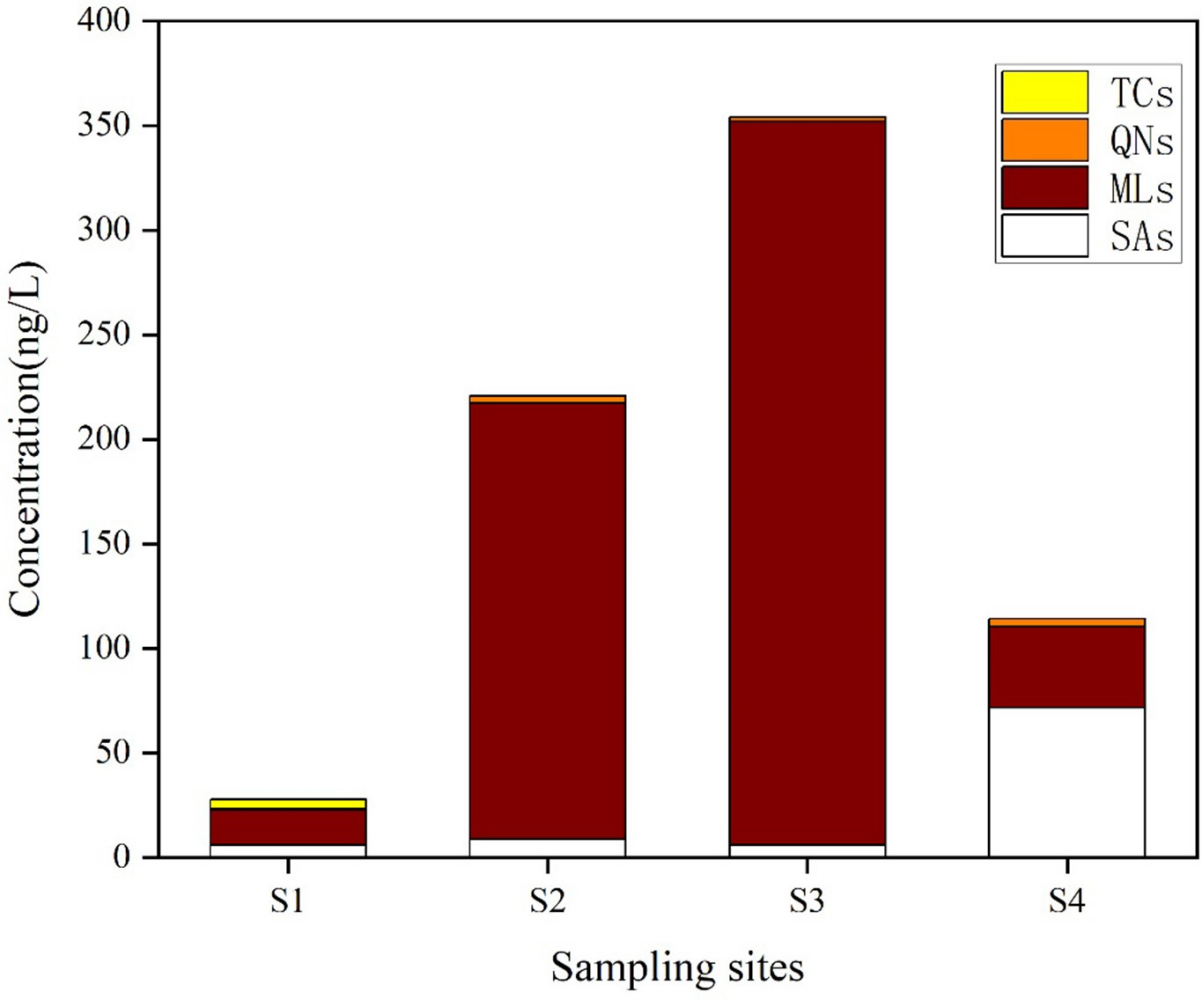
Concentrations of detectable antibiotics from four categories in the sampling sites from the receiving water bodies of four PMFs. Antibiotics with a detection frequency of >70% and an average concentration of >5.00 ng/L in this study were selected to compare surface waters at home and abroad. The target antibiotic concentrations in the waters bodies around PMFs were comparable to those in other water environments.

The antibiotics were widely detected in the water samples from the receiving waters at the level of nanogram per liter. As shown in Table 3, among 17 selected antibiotics, 13 were detected in 4 surface water samples, including 6 SAs (SDZ, SMX, TMP, SMZ, SMZ, and SMM), 4 MLs (CTM, ERY-H_2_O, LIN, and ROX), 2 FQs (CIP and OFL), and 1 TC (MTC). The total concentration range of 13 antibiotics in surface water samples was 27.88–353.98 ng/L. As shown in Fig 4, it was obvious that MLs had the highest concentration, with an average concentration of 152.46 ng/L, followed by SAs, QNs, and TCs, with an average concentration of 23.17, 2.41, and 1.15 ng/L, respectively. The average concentration of these four MLs was 86.55 ng/L, 32.09 ng/L, 22.84 ng/L, and 10.98 ng/L, respectively. In surface water samples, ERY-H_2_O (100%) was present with the highest detection frequency among four MLs; the detection frequency of CTM, LIN, and ROX was more than 50% (Table 4). Studies have shown that ERY-H_2_O and ROX are the most commonly detected MLs in the environment of our country [47], while CTM is the most common MLs in Europe and Canada [48], indicating that different types of antibiotics were used in each country and region. In addition, the pH of surface water samples was usually between 7.5 and 8.5. MLs were stable under neutral conditions where they were not easily decomposed [49], which might be another reason for the high detection concentration of such antibiotics.

**Table 4:** Concentrations of antibiotics detected in the receiving waters of PMFs.

Compounds	Frequency (%)	Concentration (ng · L^-1^)			
		Med.	Max.	Min.	Ave.
CTM	0.5714	32.25	119.56	nd^a^	32.09
ERY-H_2_O	1.0000	73.92	237.49	11.4400	86.55
LIN	0.7143	8.56	105.06	nd	22.84
OFL	0.7143	2.62	5.98	nd	3.18
ROX	0.7857	24.53	30.3900	nd	10.98
SCP	0.4286	7.145	17.7600	nd	8.43
MTC	0.2500	5.9100	6.4000	nd	5.87
SDZ	0.4286	1.11	1.54	nd	1.43
SDM	0.2143	2.56	2.78	nd	2.57
SMZ	0.7143	1.43	1.83	nd	1.69
SMX	1.0000	4.4700	59.99	nd	13.91
SMM	0.4286	4.48	9.16	nd	4.745
TMP	0.7857	2.1600	5.2000	nd	3.10

^a^ Not detected.

Moreover, SMX was found with the highest detection frequency among SAs, and its average concentration was 13.91 ng/L. The concentration of SMX in the water environment was higher than that of other SAs, which was closely related to its large-scale use in treating bacterial infections [50]. OFL was found with the highest detection frequency among two FQs, and its average concentration was 2.03 ng/L. Compared with macrolides and sulfonamides antibiotics, the low detection concentration of FQs might be related to their easy adsorption on the surface of solid particles and easy photolysis in surface water. The detection frequency of TCs in the surface water samples in this study was only 25%, and the average concentration was 1.15 ng/L. Indeed, the TCs and MLs administrated by humans were nearly two times higher than the SAs antibiotics in China [51]. The low detection frequencies and concentrations of TCs might be attributed to different medical prescription patterns in different cities and regions.

In addition, different levels of pollution were detected in four sampling points of surface waters around the PMFs (Fig 4). The highest contamination level of all the selected antibiotics was found in the receiving water of PMF3 (S3), followed by the receiving water of PMF2 (S2). However, the lowest level of pollution was found in the Hutuo river (S1), which was the receiving water of PMF1.

### Interrelationships between selected antibiotics in effluents and receiving water bodies

PMFs could not remove all antibiotics from influents, thus releasing antibiotics into the environment and then spreading in rivers. In general, the concentrations of antibiotics in receiving water bodies were lower than those in PMF effluents. A Pearson correlation analysis between the absolute concentrations of antibiotics in PMF effluents and their nearby downstream sampling sites was carried out to understand the interrelationship between antibiotics in PMFs and their receiving water bodies. Significant correlations were observed between PMF2 and S2 (*r* = 0.783, *P* = 0.016), and PMF1 and S1 (*r* = 0.792, *P* = 0.018), indicating that the emissions of these two PMFs might directly affect the content of antibiotics in receiving water bodies. No correlation was observed between PMF 4 and S4, and PMF 3 and S3. In PMF 4 and its receiving water bodies, CIP and SAs were detected only in the receiving water bodies; SAs were the main compounds, reflecting the many applications of SAs in this field. Intensive aquaculture and poultry fishing activities might be a major source of SAs. In PMF3 and its surrounding waters, antibiotics such as CTM and SMX were detected only in the samples of receiving water bodies, indicating that these antibiotics might not come from the wastewater of PMFs. The antibiotics in the receiving water bodies might also come from other pollution sources, such as urban STPs, rural wastewater, and so forth.

### Ecological risk assessment

**Fig 5:**
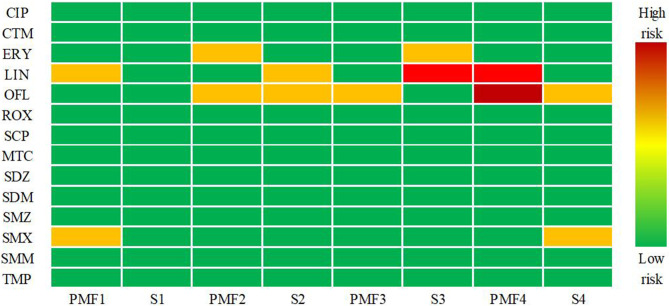
RQs for antibiotics in water samples of PMFs and receiving water bodies.

Discharge from PMFs has been identified as the main point source of antibiotics in the aquatic environment, which may pose potential ecological risks to aquatic organisms as well as potential risks to the food chain [45]. Antibiotics discharged into the environment have become a special environmental selection pressure, making the microorganisms carrying ARGs resistant and more likely to survive [52]. At the same time, ARGs replicate and spread in the environment along with the reproduction of microorganisms, posing a serious threat to human health and ecological security. Thus, the environmental risk assessment of antibiotics in the aquatic environment is necessary. The calculated RQs of antibiotics for three aquatic organisms (algae, invertebrates, and fish) are summarized in S5 Table. Algae are the most sensitive organisms in the aquatic environment to these antibiotics, which can be confirmed by other researches. By calculating RQ values in the effluent of PMFs and their receiving waters, it is concluded that the wastewaters after emission from the PMFs definitely present ecological risks. ERY-H_2_O, LIN, OFL, and SMX are all potential threats, indicating that they exert a relatively highly acute or chronic toxicological risk to aquatic organisms. As shown in Fig 5, OFL and LIN pose a high risk to algae. They showed a high ecological risk for algae in effluents of PMF4 with RQ values of 21.7 and 3.6, respectively. The discharge of wastewater from the effluent of PMFs into their receiving water bodies certainly causes the contamination of the aquatic environment.

The environmental risk of wastewater significantly decreases due to the dilution and sorption effect when the effluent is discharged into receiving water bodies. However, the risk posed by LIN and ERY-H_2_O in the receiving water bodies (S3) is higher than that in the effluent of PMF3, which can be attributed to other pollution sources, such as sewage wastewater treatment plants and livelock farms. Among the detected sulfonamides, only SMX posed a medium ecological risk to algae in the effluent of PMF 1 and the receiving water of PMF 4. However, other SAs such as SPD, SDZ, SDM, SMZ, SMM, and TMP posed no ecological risk to algae in all sampling sites. Among the other detected antibiotics, CIP, CTM, ROX, and MTC also posed no ecological risk in all sampling sites. Overall, 100% of the effluent samples from four typical PMFs posed medium to high ecological risk due to the high concentration of ERY-H_2_O, LIN, and OFL. Although the antibiotics undergo a certain degree of dilution when they are discharged into surface water, antibiotic contamination occurs in the receiving waters due to the continuous discharge of wastewater. The environmental risk of most antibiotics is low. However, the long-term drainage into aquatic ecosystems may lead to negative effects and thus should be tested. Moreover, the mixed effect caused by the interaction of different antibiotics may be more significant than the individual effect [53]. Therefore, further investigation is required.

## Conclusions

This study comprehensively investigated the occurrence and distribution of target antibiotics in four typical PMFs and their receiving water bodies in China, and assessed their potential ecological risks. The results showed that FQs and MLs were the main antibiotic species produced by the PMF, resulting in their high concentrations in the influent of the wastewater. The concentration of the antibiotics in effluent from PMF 3 was significantly higher than those of the other three PMFs, which can be due to his relatively large scale. The contamination levels are low on a global scale compared with previously reported data. The removal efficiencies of the individual antibiotics during the treatment varied widely, from negative removal to 100%, depending on their physicochemical properties and the wastewater treatment process used by each PMF. The overall removal efficiency of antibiotics treated by A^2^O, AO, and MBR alone were not significantly different but significantly higher than the BAF process alone. However, the concentration of antibiotics in the PMF discharge affects the concentration of antibiotics in the receiving water bodies to some extent by Pearson correlation analysis. The ecological risk evaluation showed that the targeted antibiotics (such as ERY-H_2_0, LIN, OFL and SMX) are all potentially at risk. This study reports useful results for managing antibiotics as emerging contaminants on a regional scale, and in the future, more attention would be paid to seasonal variation and continuous long-term monitoring of antibiotics.

## Supporting information

S1 Text Solid phase extraction (SPE) of wastewater samples. (PDF)

S1 Table The specific coordinates of each sampling locations. (PDF)

S2 Table Characteristics of the four investigated PMFs in Hebei, Jiangsu, Zhejiang and Guangdong province of China. (PDF)

S3 Table HPLC working conditions for quantification of the target antibiotics. (PDF)

S4 Table MS/MS measurement conditions of target antibiotics. (PDF)

S5 Table Average recoveries, limit of detection (LOD) and limit of quantification (LOQ) of seventeen target antibiotics for wastewater samples. (PDF)

S6 Table PNECs of the detected antibiotics for different living organisms in waters. (PDF)
