# Supplementary material for: Occurrence, fate, and risk assessment of antibiotics in typical pharmaceutical manufactories and receiving water bodies from different regions
Source: PLoS One. 2023 Jan 20;18(1):e0270945. doi: 10.1371/journal.pone.0270945 (PMC9858356; doi:10.1371/journal.pone.0270945)
Supplement: S1 Text — (PDF) [file pone.0270945.s001.pdf]

S1 Text Solid phase extraction (SPE) of wastewater samples

In the present study, SPE was carried out using Oasis HLB cartridge (6 mL, 500 mg, Waters) packed with a divinylbenzene/N-vinylpyrrolidone copolymer with hydrophilic-lipophilic balance property. The filtered aqueous samples (0.5 L) were pre-spiked to the final concentration of 1.0 µg/L with antibiotic stock standards before extraction, and all the experiments were carried out in duplicate. The SPE was conducted via the following procedures:

I. Add 100 µl 1.0 mg/L individual internal standard, i.e. SMX-D_4, ERY-^{13}C-D_3, TBD-D_4, CFX-D_8, SMZ-$^{13}C_6$, TMP-D_3, LIN-D_3 to 0.5 L sample

II. Adjust pH of the sample to 3.0 using 1.0 mol/L HCl (with pH variation less than 0.05)

III. Precondition of HLB cartridges with 6 mL of methanol and 6 mL of MilliQ water

IV. Load 1.0 L samples with flow rate about 3 mL/min

V. Rinse the HLB cartridge using 10 mL MilliQ water and dry it for 20 min under vacuuming, 1 h in the air

VI. Elute the HLB cartridge with 10 ml methanol and 5 mL MilliQ water

VII. Concentrate the eluant to about 200 µL under 45 °C water bath with N_2 stripping

VIII. Reconstitute the concentrated eluant to 2.0 mL using methanol/acetonitrile solution.

When this procedure was applied to the analysis of real samples, the pre-spike of antibiotic standards was omitted while other steps were kept the same
