# Supplementary material for: Occurrence, fate, and risk assessment of antibiotics in typical pharmaceutical manufactories and receiving water bodies from different regions
Source: PLoS One. 2023 Jan 20;18(1):e0270945. doi: 10.1371/journal.pone.0270945 (PMC9858356; doi:10.1371/journal.pone.0270945)
Supplement: S1 Table — (PDF) [file pone.0270945.s002.pdf]

S1 Table The specific coordinates of each sampling locations

Sample point	Sample Point Location
S1	38 °04.4196′N 114 °44.0454′E
S2	34 °39.7812′N 119 °12.2592′E
S3	28 °41.4072′N 121 °32.9345′E
S4	23 °41.1601′N 113 °03.5161′E
PMF1	38 °01.4344′N 114 °40.7202′E
PMF2	34 °40.4655′N 119 °12.3371′E
PMF3	28 °41.8333′N 121 °33.0358′E
PMF4	23 °40.4221′N 113 °04.4041′E
