# Supplementary material for: Occurrence, fate, and risk assessment of antibiotics in typical pharmaceutical manufactories and receiving water bodies from different regions
Source: PLoS One. 2023 Jan 20;18(1):e0270945. doi: 10.1371/journal.pone.0270945 (PMC9858356; doi:10.1371/journal.pone.0270945)
Supplement: S2 Table — (PDF) [file pone.0270945.s003.pdf]

S2 Table Characteristics of the four investigated PMFs in Hebei, Jiangsu, Zhejiang and Guangdong province of China.

PMF-No.	Daily flow (m^3)	Secondary treatment
1	7000	A_2O
2	500	MBR
3	1500	AO
4	4000	BAF
