# Supplementary material for: Occurrence, fate, and risk assessment of antibiotics in typical pharmaceutical manufactories and receiving water bodies from different regions
Source: PLoS One. 2023 Jan 20;18(1):e0270945. doi: 10.1371/journal.pone.0270945 (PMC9858356; doi:10.1371/journal.pone.0270945)
Supplement: S3 Table — (PDF) [file pone.0270945.s004.pdf]

S3 Table. HPLC working conditions for quantification of the target antibiotics

Parameter	SAs, FQs, MLs, TCs ESI+
Column temperature (℃)	40
Flow rate (mL/min)	0.30
Mobile phase A	0.2%Formic acid and 2 Mm ammonium acetate
Mobile phase B	Acetonitrile
Elution gradient	0 min: 10% B<br>0~5 min: 15% B<br>5~7 min: 20% B<br>7~11 min: 40% B<br>11~15 min: 60% B<br>15~16 min: 95% B<br>16~25 min: 95% B
