# Supplementary material for: Occurrence, fate, and risk assessment of antibiotics in typical pharmaceutical manufactories and receiving water bodies from different regions
Source: PLoS One. 2023 Jan 20;18(1):e0270945. doi: 10.1371/journal.pone.0270945 (PMC9858356; doi:10.1371/journal.pone.0270945)
Supplement: S4 Table — (PDF) [file pone.0270945.s005.pdf]

S4 Table. MS/MS measurement conditions of target antibiotics

Antibiotics	ESI	Precursor Ion (m/z)	Precursor Ion (m/z)	Declustering Potential (V)	Collision Energy (V)
SDZ	+	251.1	156.0/92.1	90	20
SMZ	+	279.1	186.2/108.0	80	23
SMX	+	254.1	156.2/108.0	90	21
SDM	+	311.3	156.0/92.1	90	22
SPD	+	250.1	156.0/108.0	90	35
SMR	+	265.1	156.0/108.0	90	23
SCP	+	285.1	156.0/108.0	90	22
SMM	+	281.3	156.0/92.1	100	36
TMP	+	291.4	156.0/123.1	100	26
TMP-D_3	+	294.4	156.3/123.1	100	28
SMX-D_4	+	258.0	160.0/92.1	90	20
SMZ-$^{13}C_6$	+	284.3	92.1/108.0	90	40
CIP	+	332.2	288.2/245.2	100	32
LIN	+	407.5	340.1/126.1	80	30
NFX	+	320.1	276.1/220.5	100	35
OFL	+	362.2	318.2/261.2	100	38
CFX-D_8	+	340.1	296.3/249.2	100	25
MTC	+	443.1	425.9/201.0	100	24
TBD-D_4		449.1	414.2/431.2	100	22
CTM	+	749.1	158/590	120	25
ERY	+	734.4	157.9/576.0	120	40
ROX	+	837.0	579/158	120	20
ERY-^{13}C-D_3	+	740.4	163.9/582.1	120	34
