# Supplementary material for: Occurrence, fate, and risk assessment of antibiotics in typical pharmaceutical manufactories and receiving water bodies from different regions
Source: PLoS One. 2023 Jan 20;18(1):e0270945. doi: 10.1371/journal.pone.0270945 (PMC9858356; doi:10.1371/journal.pone.0270945)
Supplement: S5 Table — (PDF) [file pone.0270945.s006.pdf]

S5 Table. Average recoveries, limit of detection (LOD) and limit of quantification (LOQ) of seventeen target antibiotics for wastewater samples

Antibiotics	Raw influent		
	Average recoveries (%)	LOD (ng/L)	LOQ (ng/L)
SDZ	69.1±5.8	0.39	1.29
SMZ	95.9±9.8	0.31	1.03
SMX	96.0±6.2	0.29	0.96
SDM	100.7±11.6	0.57	1.87
SPD	74.2±3.8	0.30	0.98
SCP	98.0±5.1	0.71	2.33
SMM	123.4±13.6	0.21	0.7
TMP	98.8±7.9	0.25	0.82
CTM	91.4±13.7	0.52	1.73
ERY	108.2±11.9	2.2	1.02
LIN	99.6±7.2	1.02	3.37
ROX	106.6±9.7	0.21	0.7
CIP	108.1±17.3	1.1	3.63
NFX	87.3±9.9	0.89	2.93
OFL	133.1±5.8	0.29	0.96
MTC	80.4±7.3	1.58	5.22
