# Supplementary material for: Occurrence, fate, and risk assessment of antibiotics in typical pharmaceutical manufactories and receiving water bodies from different regions
Source: PLoS One. 2023 Jan 20;18(1):e0270945. doi: 10.1371/journal.pone.0270945 (PMC9858356; doi:10.1371/journal.pone.0270945)
Supplement: S6 Table — (PDF) [file pone.0270945.s007.pdf]

S6 Table. PNECs of the detected antibiotics for different living organisms in waters

Compounds	PNECs (ng/L)		
	Freshwater algae	Invertebrates *(Daphnia)*	Fish (zebrafish embryos)
MTC	6270	30500	43800
SDZ	40390	1000	238300
SMZ	19470	700	32600
SMX	150	252000	562500
SME	28080	800	88300
SPD	20790	700	45400
SCP	28080	800	78900
SDM	9850	2259	166297
SMM	8562	2085	719037
TMP	16000	92000	100000
CTM	1400	4200	4990
ERY	2300	940	10000000
LIN	70	7200	1040222
CIP	5000	1100	N.D.
OFL	21	530	1000000
ROX	4660	6000	23000

The acute and chronic toxicity data were derived from literatures [3-7].

PNECs was calculated from the toxicity data using an assessment factor (AF) of 1000 for acute toxicity and an AF of 100 for chronic toxicity.

N.D., no data reported.
